# Elevated atmospheric CO_2_-induced reprogramming leads to decreased seed protein and nutritional quality in forest trees

**DOI:** 10.1093/plphys/kiaf463

**Published:** 2025-09-30

**Authors:** Barbara Karpinska, Rosa Sanchez-Lucas, Andrew Plackett, A Rob MacKenzie, Christine Helen Foyer

**Affiliations:** School of Biosciences, College of Life and Environmental Sciences, University of Birmingham Edgbaston, Birmingham B15 2TT, UK; Birmingham Institute of Forest Research, University of Birmingham Edgbaston, Birmingham B15 2TT, UK; School of Biosciences, College of Life and Environmental Sciences, University of Birmingham Edgbaston, Birmingham B15 2TT, UK; Birmingham Institute of Forest Research, University of Birmingham Edgbaston, Birmingham B15 2TT, UK; School of Biosciences, College of Life and Environmental Sciences, University of Birmingham Edgbaston, Birmingham B15 2TT, UK; Birmingham Institute of Forest Research, University of Birmingham Edgbaston, Birmingham B15 2TT, UK; School of Biosciences, College of Life and Environmental Sciences, University of Birmingham Edgbaston, Birmingham B15 2TT, UK; Birmingham Institute of Forest Research, University of Birmingham Edgbaston, Birmingham B15 2TT, UK; School of Biosciences, College of Life and Environmental Sciences, University of Birmingham Edgbaston, Birmingham B15 2TT, UK

## Abstract

While the global increases in atmospheric CO_2_ levels have had a beneficial effect on plant growth, the negative impacts of this CO_2_ fertilization effect on seed quality are often overlooked. Using data from acorns produced by mature oak (*Quercus robur*) trees in the eighth year of eCO_2_, we present evidence of negative consequences for seed quality. The acorns produced by the near-200-yr-old oak trees under eCO_2_ at the free air carbon dioxide enrichment facility at the Birmingham Institute for Forest Research (BIFoR) had higher phytate levels but decreased protein content. Quantitative label-free proteomics identified 335 proteins in all acorns, but 9 proteins were undetectable in acorns produced under eCO_2_ compared to ambient air (aCO_2_), and 1 protein was uniquely detected in the eCO_2_ acorns. Further subsets of proteins were identified with either higher or lower abundance in eCO_2_ than aCO_2_ acorns. Proteins that were more abundant in the acorns produced under eCO_2_ include allene oxide cyclase and phosphomannomutase. RNA-seq analysis revealed that 154 transcripts were more abundant in the eCO_2_ acorns compared to those grown under aCO_2_, while 54 were much less abundant. Transcripts encoding several transcription factors and phytohormone signaling proteins, as well as trehalose 6-phosphate phosphatase, were increased in eCO_2_ acorns. Taken together, these findings demonstrate that the transcriptome and proteome profiles of acorns produced under eCO_2_ are significantly changed compared to those produced in aCO_2_, with important implications for seed metabolism, particularly those underpinning the observed changes in seed protein and phytate levels.

## Teaser

Growth under elevated CO_2_ rewired the proteome and transcriptome profiles of acorns produced by 200-yr-old oak trees, resulting in lower seed protein contents but higher phytate levels.

## Introduction

Carbon dioxide (CO_2_) is the essential substrate for photosynthesis. The global increases in atmospheric CO_2_ levels over the last hundred years have significantly increased photosynthetic CO_2_ assimilation rates (Leakey 2009; Bernacchi and VanLoocke 2015). This “CO_2_ fertilization effect” increases the ability of terrestrial ecosystems to serve as a sink for anthropogenic CO_2_ emissions (Pan et al. 2011; Friedlingstein et al. 2023) under the assumption of sustained regenerative capacities of forests. However, there is evidence that plants optimize photosynthesis under elevated CO_2_ (eCO_2_) conditions, with C_3_ plants showing a decrease in the abundance of the ribulose-1,5-bisphosphate carboxylase/oxygenase (Rubisco) protein and other photosynthetic proteins (Ancín et al. 2024). The acclimation of photosynthesis to eCO_2_ is more pronounced when environmental and/or genetic factors limit sink strength (Tausz-Posch et al. 2020; Hu et al. 2022), and it is hypothesized that optimization occurs to maintain high rates of photosynthesis with the lowest possible nutrient use (Smith and Keenan 2020). Lower photosynthetic demand for Rubisco under eCO_2_ may reduce the nitrogen content of leaves (Ainsworth and Long 2021). Moreover, the suppression of photorespiration that inevitably occurs under eCO_2_ is linked to decreased nitrate assimilation, together with lower levels of leaf and seed protein (Rachmilevitch et al. 2004; Myers et al. 2014). The integration of photorespiration and nitrogen assimilation may also provide a partial explanation for the observed decreases in seed protein (Shi and Bloom 2021). However, the effects of increasing atmospheric CO_2_ on wider plant signaling and the regulation of nutrient acquisition and partitioning are complex and poorly understood (Foyer et al. 2025a, 2025b).

Tree seeds and nuts are essential to forest food webs (Ickowitz et al. 2022) and comprise an increasing component of human diet (Balakrishna et al. 2022). Increased atmospheric CO_2_ levels are predicted to reduce the nutritional quality of seeds and fruit, based on laboratory and free air carbon dioxide enrichment (FACE) experiments (Fernando et al. 2012; Taub and Wang 2013; Fernando et al. 2014; Loladze 2014; Myers et al. 2014; Dong et al. 2018; Zhu et al. 2018). For example, growth under eCO_2_ decreased the concentration of 25 important minerals by 8% on average, and the ratio of carbohydrates to minerals was increased in these plants (Loladze 2014). While lower leaf protein nitrogen content may also provide less nitrogen to seeds and fruits (Taub and Wang 2013), the observed decreases in seed nutrients and protein do not appear, as yet, to have adversely affected seed germination.

Micronutrients such as Fe and Zn are essential to both plant and animal health. While these micronutrients can be accumulated to high levels in seeds, their bioavailability in the diet remains problematic. This is largely because common metabolites such myo-inositol hexaphosphate (InsP6) and polyphenols (PPs) limit the bioavailability of Fe and Zn in seeds (Gibson et al. 2018). Phytic acid and its salts, which are together called “phytate,” are potent inhibitors of iron and zinc absorption. Phytate is a major phosphate store in seeds of species such as *Arabidopsis* and common bean, the levels of phytate being intrinsically linked to the availability of phosphate (Bouain et al. 2022). The ability to restrict phosphate import into chloroplasts is an important mechanism in limiting eCO_2_-dependent increases in phytate and hence nutrient bioavailability (Bouain et al. 2022). However, much remains uncertain about how this regulation contributes to overall seed phytate levels.

This negative impact of eCO_2_ thus has the potential to decrease global availability of essential nutrients and increase human malnourishment (Smith and Myers 2018; Weyant et al. 2018; Beach et al. 2019), not least because the mineral content of maize and sugarcane was also reduced in eCO_2_. A previous study on the effects of eCO_2_ on seed production in pine trees (*Pinus taeda*) growing in the Duke Forest FACE experiment showed that the number of mature, viable seeds doubled in the high-CO_2_ plots over 10 years, but there was no CO_2_ effect on mean seed mass, viability, or the seed carbon-to-nitrogen ratios (Way et al. 2010). This study concluded that unlike many crops, woody plants may benefit from future atmospheric CO_2_ by producing larger numbers of seeds without suffering degraded seed quality (Way et al. 2010). Moreover, a meta-analysis of the effects of 9 years of growth under eCO_2_ on a scrub-oak community of plants and herbivores at the Kennedy Space Center, Florida (United States) revealed a negative impact on herbivores in every year under eCO_2_ compared to ambient CO_2_ (Stiling and Cornelissen 2007). This analysis showed that eCO_2_ significantly decreased a range of key insect parameters such as herbivore abundance, development time, relative growth rates, and pupal and increased relative consumption rates. Such findings suggest that growth under eCO_2_ has a negative impact on the nutritional quality of the oak tissues, which form the basis of insect food, compared to ambient eCO_2_.

Seeds are provided with essential carbohydrates, carbon metabolites, and amino acids that are predominantly synthesized de novo in the surrounding aerial tissues (Pirredda et al. 2024). The remobilization of stored nutrients can contribute during seed filling (Salon et al. 2001; Sperotto et al. 2014), but essential micronutrients are also acquired from the soil and transported to the developing organs (Pirredda et al. 2024). To our knowledge, the mechanisms by which growth under eCO_2_ impacts seed nutritional quality in perennial trees (and tree crops) have not been investigated. Tree crops are an important and economically valuable component of global agriculture (Molnar et al. 2013). An ongoing FACE experiment on mature oak (*Quercus robur* L.; Hart et al. 2020) has demonstrated a similar CO_2_ fertilization effect as in annual plants, with a 25% increase in photosynthesis under eCO_2_ compared to the trees growing in ambient air (aCO_2_) (Gardner et al. 2022a,b, 2023), a decrease in the “water cost of carbon gain” (reduced by ∼40%) (Gardner et al. 2022a,b, 2023), greater fine root production (Ziegler et al. 2023), and enhanced release of root exudates (Norby et al. 2024) into the surrounding soil for priming nutrient acquisition to balance the accumulation of nitrogen, phosphorus, and other essential minerals with increased carbon capture. A significant effect on fertility was also detected, with an increased number of flowers beginning (but not completing) acorn development (McClory et al. 2024). In contrast to annual plants, however, leaf nitrogen content was maintained under eCO_2_ (Gardner et al. 2022a,b, 2023). Furthermore, the relative contributions of direct nutrient synthesis/transport and remobilization of stored reserves have not been quantified during seed filling of trees. With their perennial growth habit and typically slower seed maturation process (Pirredda et al. 2024), trees might be expected to possess larger reserves of stored nutrients compared to annual plants, and so remobilization could potentially play a greater role during seed filling than in annual plants. As such, the effect of eCO_2_ on seed and fruit nutritional quality from perennial trees remains unknown and hard to predict. To address this knowledge gap directly, we explored the effects of eCO_2_ on the quality of mature acorns compared to those produced under ambient conditions at the same site.

The following study was conducted to determine the effects of long-term exposure to eCO_2_ (7 to 8 years) on the protein content and composition of mature acorns that were produced by 180-yr-old oak trees that had been growing either under aCO_2_ or under eCO_2_ conditions at the FACE site (Foyer et al. 2025a). In addition, we analyzed eCO_2_-dependent effects on the transcriptome profile of the acorns produced during the 7th and 8th years of eCO_2_ treatment, and we documented the effects of eCO_2_ on the micronutrient status of the seeds. Our overarching goal was to establish how growth under eCO_2_ altered the nutritional quality of acorns and to determine the molecular and molecular mechanisms and drivers of the observed responses to environmental change.

## Results

**Figure 1.**
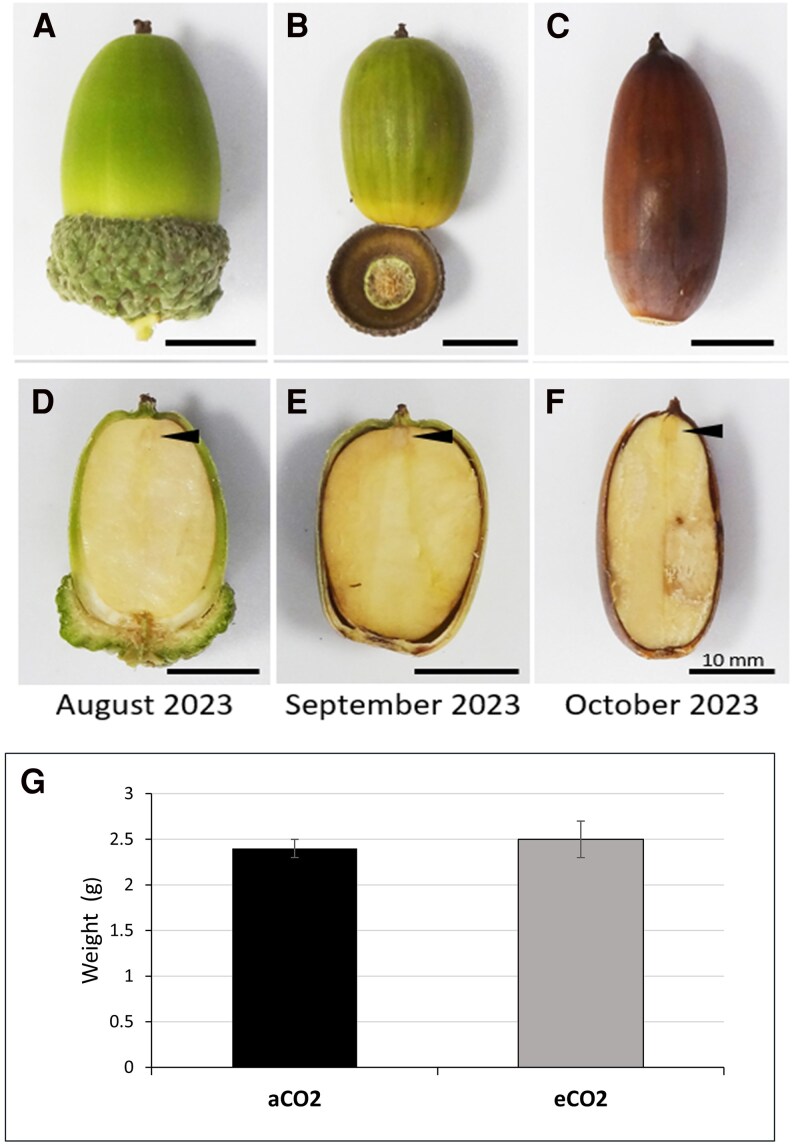
The physiology of acorns produced under eCO_2_ and aCO_2_. Representative images of the range of acorn developmental stages harvested. The harvested acorns were approximately at equivalent to stages 7 and 8 in Miguel et al. (2015). Whole acorns **A** to **C)** and cut-throughs of the same acorns **D** to **F)** showing the embryo indicated by arrowheads. The average fresh weight of acorns **G)**. Error bars represent the mean plus or minus the standard error of over 20 acorns per sample.

Acorns were collected from 180-yr-old oak trees growing under eCO_2_ conditions during the 7th and 8th years of eCO_2_ treatment. Over the whole period of eCO_2_ exposure, the trees growing under eCO_2_ conditions were found to produce significantly larger acorns, except for in nonmast years where no differences in acorn size were observed. The acorns that were selected for the present analysis had a similar weight and size to those growing under aCO_2_ (Fig. 1), to avoid possible dilution effects. No differences in seed germination rates or viability were observed. In all cases, germination rates were between 97% and 99%.

**Figure 2.**
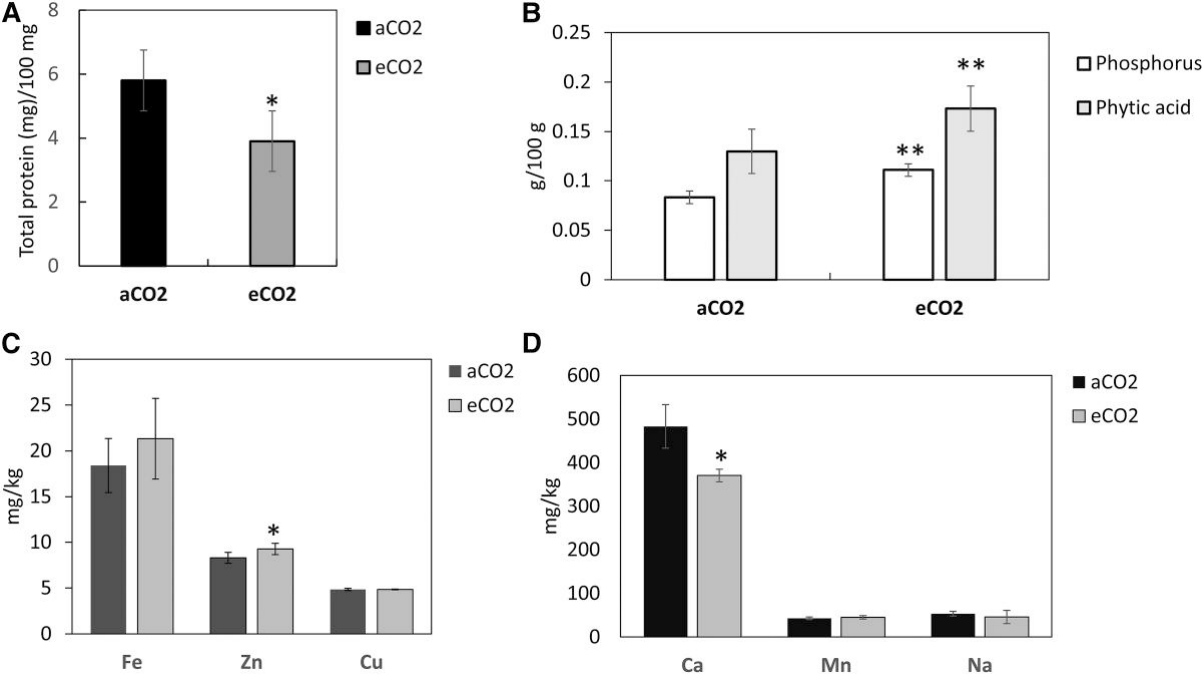
A comparison of the protein and mineral levels in the acorns from the 200-yr-old oak trees grown either in aCO_2_ or eCO_2_. Total protein in milligram per 100 mg fresh weight **A)**; phosphorus and phytic acid levels in milligram per 100 mg fresh weight **B)**; Fe, Zn, and Cu levels in milligram per kilogram fresh weight **C)**; Ca, Mn, and Na levels in milligram per kilogram fresh weight **D)**. Error bars represent the mean plus or minus the standard error of over 4 acorns per sample, with 4 replicate samples per experiment. The asterisks denote statistical significance (* **<** 0.05; **< 0.01) using a standard T-test.

The acorns produced under eCO_2_ had significantly less protein than those growing under aCO_2_ (Fig. 2A). Although the eCO_2_ acorns accumulated significantly more phosphorus and phytate than aCO_2_ controls (Fig. 2B), they had similar levels of Fe, Zn, Cu, Mn, and Na (Fig. 2C and D). Of the minerals that were detected, only the levels of calcium were significantly lower in the eCO_2_ acorns than those grown under ambient CO_2_ (Fig. 2D). These results contrast with those of a past meta-analysis across annual crop plants, where Fe, Zn, and Cu were significantly reduced under eCO_2_ (Loladze 2014). The eCO_2_ acorns accumulated significantly less protein, which is consistent with data from previous studies (Loladze 2014).

**Figure 3.**
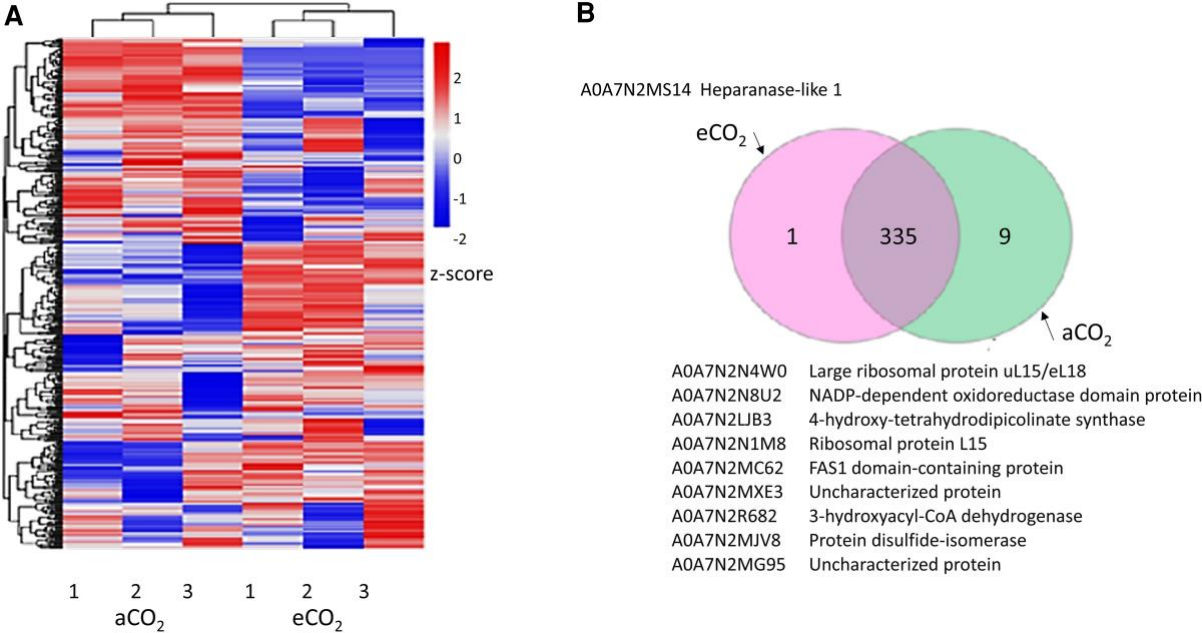
The profiles of the whole proteome of acorns produced under aCO_2_ and eCO_2_. Heatmap comparisons of the proteomes of acorns grown either in aCO_2_ with or eCO_2_  **A)**, showing 3 biological replicates (1 to 3) in each case. Venn diagram of the proteins that were detected only in acorns produced under eCO_2_, under both growth conditions, or only in acorns produced under eCO_2_  **B)**.

Label-free quantitative proteomics analysis and RNA-seq comparisons revealed that, as well as a quantitative difference in total protein, under eCO_2_ treatment, acorns had a significantly different proteome and transcriptome composition compared to the aCO_2_ control (Supplementary Fig. S1). The label-free shotgun proteomics analysis of acorns from each treatment (Fig. 3A) identified 335 proteins present in all samples (Supplementary Table S1). Nine proteins were found only in aCO_2_ acorns. These include proteins involved in metabolism, defense, and ribosomal proteins (Fig. 3B). Additionally, 1 protein known as antisense to ERCC-1 (ASE-1), associated with organizer regions of chromosomes during cell division (Whitehead et al. 1997), was uniquely detected in eCO_2_ acorns (Fig. 3B).

**Figure 4.**
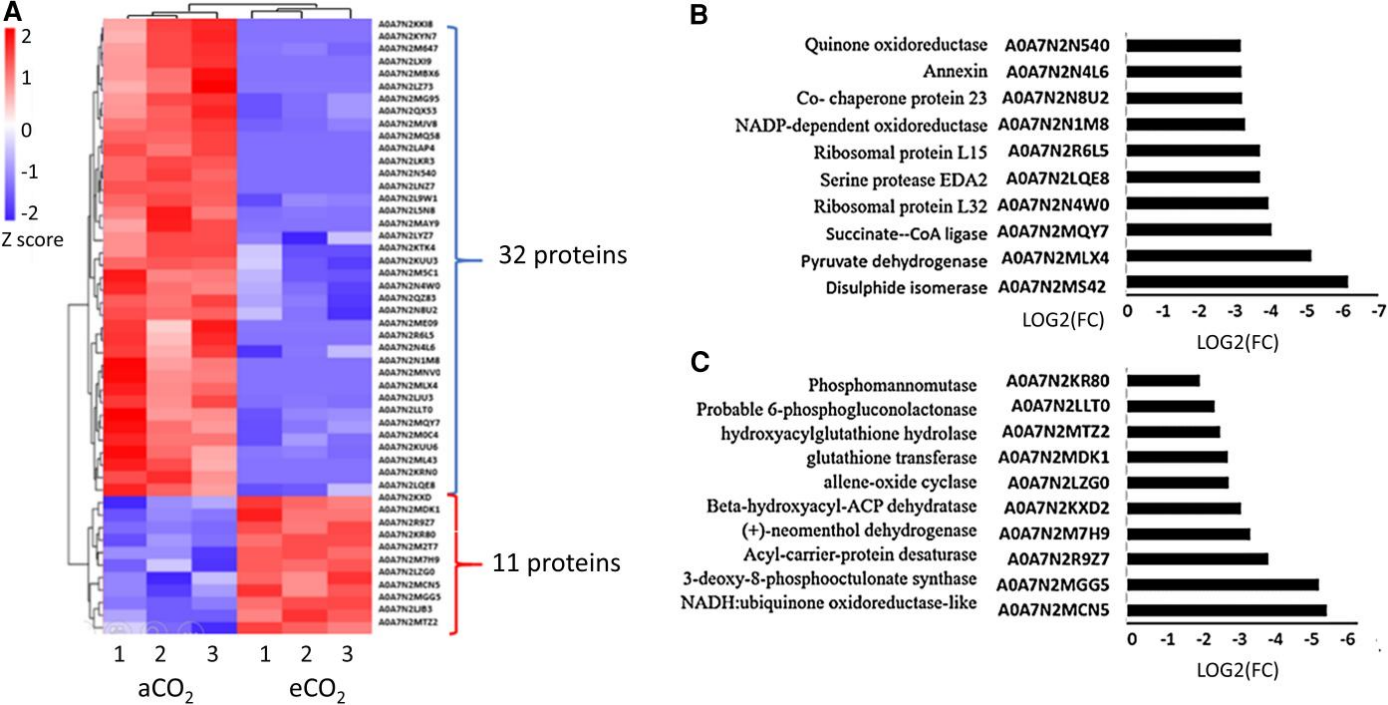
The profiles of the proteins that were the most changed in abundance in the acorns produced under aCO_2_ and eCO_2_. Heatmap comparisons of the proteins that were most changed in abundance in the acorns grown either in aCO_2_ with or eCO_2_  **A)**, showing 3 biological replicates (1 to 3) in each case The 10 most abundant proteins in eCO_2_ relative to aCO_2_  **B)**. The 10 most decreased proteins in eCO_2_ relative to aCO_2_  **C)**. Statistical significance was determined using a 2-sample *t*-test with the Benjamini–Hochberg correction, where FC represents the fold change ratio (eCO_2_/aCO_2_).

A subset of 32 proteins showed a lower abundance in the eCO_2_ acorns than in those from aCO_2_ (Fig. 4A; Supplementary Table S2), whereas 11 proteins were more abundant under eCO_2_ (Fig. 4A). The proteins that were most decreased in abundance include pyruvate dehydrogenase subunits, succinate-coenzyme A ligase, and an alcohol dehydrogenase (Fig. 4B). Of the proteins that were less abundant under eCO_2_, many are involved in metabolism (Supplementary Table S2). Examples are fructose-bisphosphate aldolase, which plays a key role in glycolysis and gluconeogenesis, catalyzing the conversion of fructose 1,6-bisphosphate to glyceraldehyde 3-phosphate and dihydroxyacetone phosphate, and malate dehydrogenase, which catalyzes the reversible conversion of malate to oxaloacetate in the tricarboxylic acid (TCA) cycle (Supplementary Table S2). Other less abundant proteins in eCO_2_ acorns include serine carboxypeptidase-like acyltransferases (Supplementary Table S2), which contribute to the regulation of plant growth, development, and stress responses. These enzymes catalyze transacylation reactions involving energy-rich 1-O-β-glucose esters in the synthesis of a diverse range of secondary compounds (Yao et al. 2022). Moreover, under eCO_2_ acorns had less methylenetetrahydrofolate dehydrogenase/methyltetrahydrofolate cyclohydrolase (MTHFD1), which is an important enzyme in 1-carbon metabolism (Supplementary Table S2). This bifunctional enzyme also fulfills essential roles in the epigenetic networks of plants such as *Arabidopsis* (Groth et al. 2016). Proteins that were more abundant in the acorns produced under eCO_2_ (Fig. 4C; Supplementary Table S2) include allene oxide cyclase, which is a key enzyme in the biosynthesis of jasmonic acid (JA), and phosphomannomutase, which is a key enzyme of the mannose metabolic pathway that is important in the synthesis of GDP-mannose, required for protein glycosylation (Liang et al. 2024). Taken together, these findings suggest a switch from carbon reduction to the carbon oxidation pathways that support plant defense, as well as increase in secondary metabolism.

**Figure 5.**
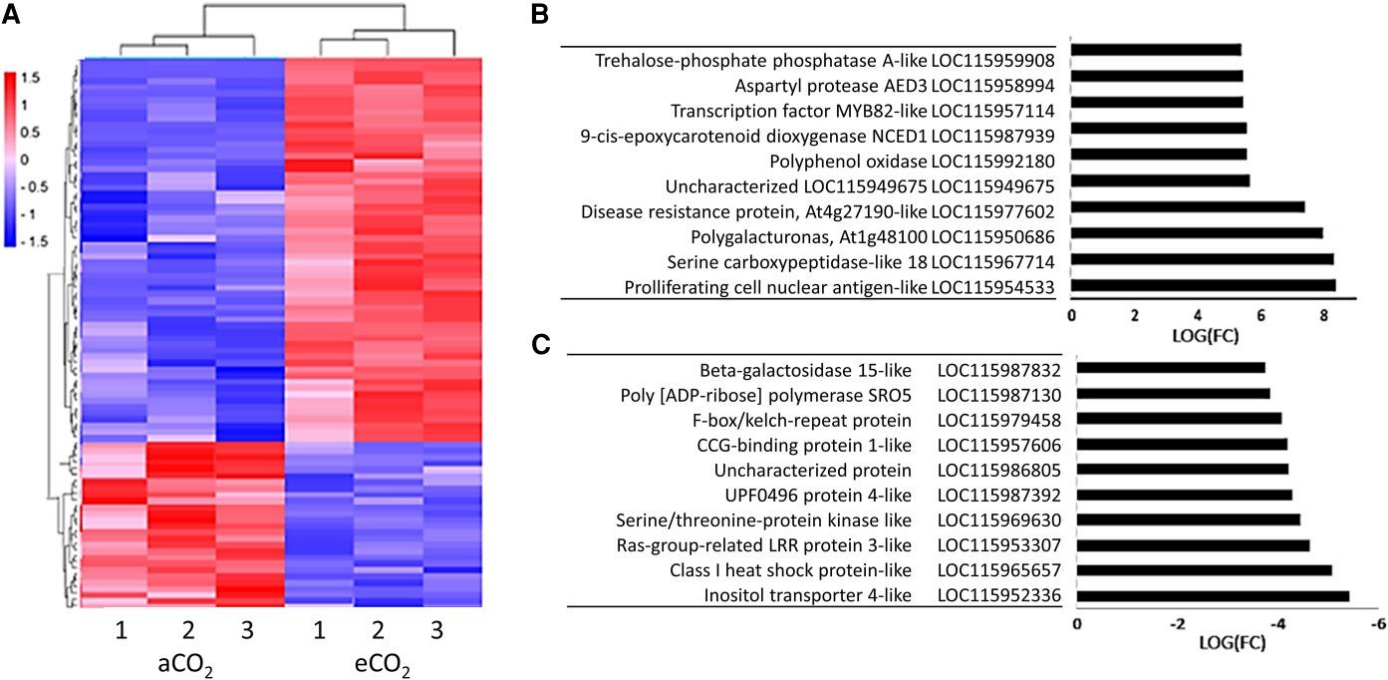
The profiles of the transcripts that were the most changed in abundance in the acorns produced under aCO_2_ and eCO_2_. Heatmap comparisons of the transcripts that were most changed in abundance in the acorns grown either in aCO_2_ with or eCO_2_  **A)**, showing 3 biological replicates (1 to 3) in each case. The 10 most abundant transcripts in eCO_2_ relative to aCO_2_  **B)**. The 10 most decreased transcripts in eCO_2_ relative to aCO_2_  **C)**. Statistical significance was determined using a 2-sample *t*-test with the Benjamini–Hochberg correction, where FC represents the fold change ratio (eCO_2_/aCO_2_). LOC gene numbers are listed in Supplementary Table S3.

The RNA-seq analysis revealed that 154 transcripts were much more abundant in the eCO_2_ acorns compared to those grown under ambient CO_2_, while 54 were much less abundant (Fig. 5A and Supplementary Table S3). The 10 transcripts showing the greatest increases in abundance under eCO_2_ included a trehalose 6-phosphate phosphatase (Tre6P) A-like (Fig. 5C). This finding is particularly interesting because it suggests changes in the regulation of sugar metabolism and signaling (Fichtner and Lunn 2021). Trehalose acts as a central regulator of carbon and nitrogen metabolism as well as source–sink interactions that determine plant growth and stress responses (Lunn et al. 2024). Within this context, Tre6P is an essential signaling metabolite linking growth and development to plant carbon status (Fichtner and Lunn 2021), including between the parent plant and developing embryos to regulate both seed-set (Nuccio et al. 2015) and seed filling (Meitzel et al. 2021). High Tre6P levels favor decreased sucrose levels and stimulate carbon flow into organic and amino acids, via the posttranslational activation of enzymes such as phosphoenolpyruvate carboxylase and nitrate reductase (Kerbler et al. 2023). This increased abundance of transcripts encoding Tre6P phosphatase suggests a switch of carbon flow toward carbohydrates and away from organic and amino acids in the acorns produced under eCO_2_.

An examination of the 10 transcripts that were most decreased in the eCO_2_ acorns reveals modifications that suggest altered regulation of proteins involved in cell signaling such as a serine/threonine proteinase, heat shock protein-like, and the poly(ADP-ribose) polymerase (PARP) SRO5 (Fig. 5C). There is also a marked decrease in transcripts encoding an inositol transporter 4-like protein. Inositol transporters (INTs), which localize to the plasma membrane or tonoplast membrane, are responsible for the transport of myo-inositol, which plays crucial roles in plant growth, development and stress responses (Fig. 5C). The *Arabidopsis* AtINT4 protein is a highly specific proton symporter for myo-inositol that is strongly expressed in pollen and phloem companion cells (Schneider et al. 2006).

**Figure 6.**
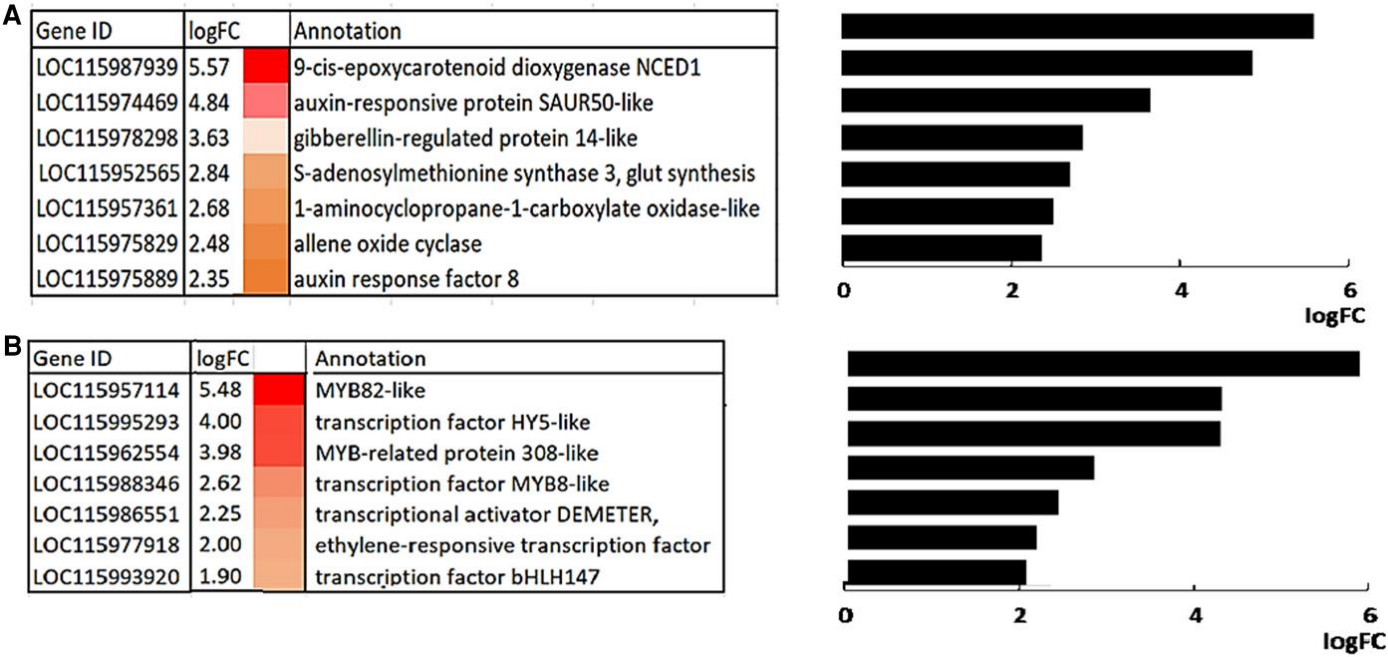
The transcripts encoding TFs and proteins associated with hormone metabolism that were the most changed in abundance in the acorns produced under aCO_2_ and eCO_2_. Hormone-related transcripts **A)** and TF transcripts **B)** that were more abundant in the acorns grown under eCO_2_ than aCO_2_ logFC.

Transcripts involved in the metabolism and signaling of phytohormones such auxin, JA, and gibberellins were increased in the eCO_2_ acorns (Fig. 6A). For example, the increase in transcripts encoding 9-cis-epoxycarotenoid dioxygenase NCED1 under eCO_2_ suggests an increase in the pathway of ABA synthesis (Fig. 6A). ABA plays a crucial role in seed maturation and the onset of dormancy, acting in antagonism to ethylene (Ali et al. 2022). In addition, there was a significant increase in transcripts encoding S-adenosylmethionine synthase 3 (Fig. 6A). S-adenosylmethionine is an essential cofactor for methyl transfer reactions, including those involved in the biosynthesis of plant hormones and the methylation of DNA, RNA, and histones.

In addition, transcripts encoding several transcription factors (TFs) showed an increased abundance in the acorns produced under eCO_2_ (Fig. 6B), including MYB308 that regulates the expression of genes involved in lignin biosynthesis and iron uptake (Fan et al. 2022) and MYB8, which is involved in plant responses to stress, particularly iron deficiency regulating iron uptake and translocation, promoting chlorophyll accumulation under iron deficiency (Gong et al. 2024). MYB8 participates in iron homeostasis by regulating iron uptake and the expression of translocation-related genes, including Fe deficiency-induced transcription factor (*FIT*), iron-regulated transporter (*IRT1*), and *nicotianamine synthase 4* (*NAS4*). Other TFs that are more abundant in acorns produced under eCO_2_ include MYB82, which is a nuclear-localized transcription activator that regulates trichome development in *Arabidopsis thaliana* (Liang et al. 2014); bHLH147, which is a basic helix-loop-helix (bHLH) TF involved in cell proliferation and differentiation in a range of developmental processes, as well as other processes such as shade avoidance (Buti et al. 2020); *HY5*, which helps to establish seed dormancy (Chen and Xiong 2008), and *DEMETER*, which encodes a DNA glycosylase that is essential for seed viability and gene imprinting, regulating seed endosperm proliferation in *Arabidopsis* (Choi et al. 2002). Collectively, these results are thus consistent with an effect of eCO_2_ on acorn nutrient storage and composition. Gene Ontology (GO) term enrichment analysis of differentially expressed genes (DEGs; Supplementary Fig. S2A) and proteins (DEPs; Supplementary Fig. S2B) confirmed significant changes in proteins with key molecular functions and biological processes relating to these processes.

## Discussion

Elevated atmospheric CO_2_ levels have a positive impact on the growth of C_3_ plants, because of increased photosynthesis, carbon gain, and organ carbohydrate availability. However, the resulting impact on essential minerals and nutrients, together with lower leaf and seed protein contents in crop plants, are a cause for concern (Fernando et al. 2012; Taub and Wang 2013; Fernando et al. 2014; Loladze 2014; Myers et al. 2014; Dong et al. 2018; Zhu et al. 2018). While the adverse effects of eCO_2_ on the accumulation of proteins and minerals in essential food crops are widely recognized, the mechanistic basis for these responses remains poorly understood, and there is limited information concerning the responses on perennial plants growing in seminatural environments. We therefore studied the acorns produced by mature (∼180-yr-old) oak trees at the BIFoR FACE facility, which is the only ongoing forest FACE facility in the northern hemisphere (Norby et al. 2016; Hart et al. 2020).

The acorns analyzed in the present study were collected in the 7th and 8th years of eCO_2_ treatment. The trees growing under eCO_2_ conditions produce significantly larger acorns in mast years but not in nonmast years (manuscript in preparation). A previous study in pine (*P. taeda*) trees growing in the Duke Forest FACE site (Way et al. 2010) reported a doubling in the number of mature, viable seeds in the eCO_2_ plots over 10 years, but there was no eCO_2_effect on mean seed mass, viability, or the seed carbon-to-nitrogen ratios. Such differences in seed responses to growth under eCO_2_ are likely to result from differences in the age of the trees, other environmental differences, and variations in the physiological/morphological limitations between species. To avoid possible dilution effects, we selected eCO_2_ acorns that had a similar weight and size to those growing under aCO_2_ for the present analysis. Our finding that the protein composition of acorns grown under eCO_2_ is distinct from that of aCO_2_ acorns is surprising, given that seed protein contents are often considered to be relatively stable compared to other organs (Marcone 1999; Adhikari et al. 2022; Yang et al. 2023a,b). The relative stability of seed proteins within a given species is due to factors such as the function of seeds as storage organs for the embryo, the protective mechanisms within seeds, and the specific regulation of seed storage protein synthesis (Yang et al. 2023a,b). While the transcriptome profiles of seed storage proteins have been extensively mapped, little information is available on the posttranscriptional, translational, and posttranslational controls that regulate seed protein accumulation. Moreover, seed protein content is a complex and unstable trait, integrating all the developmental processes (Burstin et al. 2007). Much remains uncertain about how specific triggers and environmental signals influence and direct seed protein synthesis. The data presented here show that growth under eCO_2_ not only decreases the protein content of acorns but also has a profound effect of their protein composition and transcript profile. This finding is consistent with observations in many annual crop plants, in which seed protein content is lower under eCO_2_, although in some cases, a small increase has been reported (Bhatia et al. 2021; Digrado et al. 2024). However, a qualitative assessment of protein composition has not been reported in previous studies. In contrast to annual plant studies, no significant reductions in micronutrient concentrations per se were identified in acorns under eCO_2_. The transcriptome analysis reported here reveals the increased expression of several MYB TFs involved in iron uptake and homeostasis in eCO_2_ acorns, including MYB308 that regulates the expression of genes involved in iron uptake (Fan et al. 2022) and MYB8, which regulates iron uptake and translocation in response to iron deficiency (Gong et al. 2024). These TFs may be important in responding to the perception of eCO_2_ and resultant effects on iron accumulation and bioavailability.

Although micronutrient concentrations were not affected, the bioavailability of iron and zinc may be negatively affected in eCO_2_ because of a higher accumulation of phytate, an iron chelator that is produced through stepwise phosphorylation of myo-inositol. The decreased expression of an inositol transporter 4 (INT4)-like protein in acorns produced under eCO_2_ may prevent negative effects on seed germination. Phytic acid accumulation was limited in *Arabidopsis* plants grown under eCO_2_ because of the expression of the PHT4;3 transporter, which facilitated the regulated decrease in chloroplast phosphate, and thus phytate synthesis (Bouain et al. 2022). These authors provided evidence that the regulation of chloroplast phosphate metabolism prevented increases in phytate that would otherwise have a negative impact on plant growth under eCO_2_ (Bouain et al. 2022). This study thus suggests that, while there may be similarities and differences in the seed-filling response of eCO_2_ between long-lived perennials and annual crops, increasing atmospheric CO_2_ concentration will negatively affect the nutritional quality of all seed crops irrespective of their growth habit.

The molecular mechanisms by which eCO_2_ regulates seed composition are poorly understood. While a dilution effect caused by the eCO_2_-dependent stimulation of growth is often cited as the basis for the observed decreases in nutritional quality, the acorns produced under eCO_2_ accumulated more phosphate than aCO_2_ controls, and the trees have no symptoms of nitrogen limitation (Norby et al. 2024; Foyer et al. 2025a). This finding strongly suggests that processes other than nutrient deprivation have a significant impact on the protein and nutrient quality of seeds, supporting the concept that CO_2_ signaling has important implications for plant growth and metabolism (Foyer et al. 2025b). An examination of the transcripts and proteins that were the most changed in abundance in the eCO_2_ acorns reveals that there are important shifts in metabolism, as well as in the genetic and epigenetic controls of metabolism. For example, the increase in transcripts encoding Tre6P phosphatase in the eCO_2_ acorns suggests a redirection of carbon flow.

These results reveal the strategies that plants use to combat a carbohydrate-rich diet. The data presented in this paper has both mechanistic importance and direct ecological implications for oaks and oak herbivores and potentially to increasing human plant-based diets. The results show that growth under eCO_2_ triggers nutrient deficiency responses regarding other essential elements such iron and nitrogen. The uptake and homeostasis of these elements are essential if the positive impact on the growth of C_3_ plants is balanced by nutrient accumulation, particularly in seeds. The findings present a mechanistic explanation of why the performance of insect herbivores is decreased under eCO_2_ (Stiling and Cornelissen 2007). Taken together, the data presented here show that eCO_2_ triggers an increase in seed phytate levels that may lead to decreased bioavailability of essential elements, such as iron, that are not coupled to carbon in Earth system and dynamic vegetation models, redirecting metabolism to deal with excess carbon (Medlyn et al. 2015).

## Materials and methods

### Sample collection

Recently fallen acorns were collected from the forest floor beneath the oak trees growing either in aCO_2_ or eCO_2_ at the BIFoR FACE facility, Mill Haft, Staffordshire, from 2022 to 2024. All acorns harvested were of final size and in the process of or recently completed maturation (Fig. 1). Within 24 h of collection, the acorns were sorted, with stringent quality checks for any obvious infection prior to analysis, floatation-based seed selection separating viable seeds, and finally sterilized with 2.5% sodium hypochlorite to remove any contamination (Sanchez-Lucas et al. 2023). In all cases, batches of 3 to 5 acorns were weighed and frozen at −80 °C prior to analysis. Remaining seeds were pregerminated and planted for further analysis of seedling growth and development following the protocol described by Sanchez-Lucas et al. (2023).

### Sample preparation

Whole acorns (3 to 5 per biological sample) were ground in liquid nitrogen and protein extracted using the protocol of Wang et al. (2006) with some modifications. Samples (100 mg) of acorn powder were washed twice in acetone containing TCA (10%). Pellets were resuspended in SDS buffer [1% SDS, 10 mm Tris-HCl (pH 8), and 0.5% beta-mercaptoethanol (BME)], incubated on ice prior to phenol extraction. Each phenol phase was precipitated with methanol-ammonium sulfate. Pellets were washed and resuspended in urea buffer (7 m urea, 0.1 m Tris, pH 8, 0.001 m EDTA, and 1% DTT). Protein quantity was estimated using Coomassie Brilliant Blue G-250 (Qiagen).

### Proteomics analysis

Quantitative label-free proteomics was performed using liquid chromatography-tandem mass spectrometry (LC-MS/MS) at the University of Birmingham Proteomics Facility (https://www.birmingham.ac.uk/facilities/genomics/about/proteomics). Analyses were conducted on an Q Exactive HF mass spectrometer coupled with a Dionex nanoflow liquid chromatography system. Raw data were processed with Proteome Discoverer v2.2. Only proteins identified by at least 2 unique peptides and with a minimum sequence coverage of 5% were considered for downstream analysis. Protein abundance distributions were evaluated using histograms to assess data variance. Differentially expressed proteins (DEPs) were identified via 2-sample *t*-tests with false discovery rate (FDR) correction using the Benjamini–Hochberg method (adjusted *P* < 0.05). Visualization of proteomic data, including histograms, heatmaps, principal component analysis (PCA), and volcano plots, was performed using Perseus v2.1.3.0.

### RNA extraction and sequencing

Total RNA was extracted from 4 biological replicates of acorn tissue (120 mg each) using a modified protocol based on Vennapusa et al. (2020). Ground tissue was suspended in 600 *µ*L of RNA extraction buffer containing 2% SDS, 100 mM Tris-HCl (pH 8.0), 10 mM EDTA, and 2 mM DTT. After centrifugation at 14,000 × *g* for 5 min at 4 °C, the supernatant was collected and extracted with an equal volume of phenol:chloroform:isoamyl alcohol (25:24:1). Following gentle inversion and centrifugation under the same conditions, the aqueous phase was recovered and reextracted with an equal volume of chloroform followed by brief centrifugation (15 to 20 s at 14,000 × *g*, 4 °C). RNA was precipitated with 1 volume of prechilled isopropanol in the presence of 0.3 M sodium acetate (pH 5.2) and 0.6 M NaCl. Samples were incubated at −20 °C for 20 min and centrifuged at 14,000 × *g* for 15 min at 4 °C. The resulting pellet was washed twice with 70% ethanol, each followed by centrifugation at 14,000 × *g* for 2 min at 4 °C.

Total RNA (1 *µ*g per sample) was used for cDNA library preparation, including double-stranded cDNA synthesis, end repair, poly(A) tailing, adaptor ligation, and PCR enrichment. Libraries were prepared by the University of Birmingham (ENvision Services) and sequenced by Novogene (Cambridge, United Kingdom) on the Illumina NovaSeq 6000 platform, generating 150 bp paired end reads (≥5 million reads per sample). Following quality control, reads were aligned to the *Quercus lobata* reference genome (ValleyOak3.0, Ensembl Plants) using HISAT2 (v2.2.1) with default settings and GTF-guided splice alignment. Output BAM files were used for downstream quantification.

### Bioinformatic analysis of RNAseq data

Differential gene expression analysis was conducted in R using the edgeR package. Raw counts were filtered to remove low abundance transcripts and normalized using the trimmed mean of M-values (TMM) method. A generalized linear model (GLM) was fitted, and differential expression between aCO_2_ and eCO_2_ conditions was evaluated using the likelihood ratio test (*glmLRT*). Genes with a FDR < 0.05 and absolute log_2_ fold change ≥ 1 were considered significantly differentially expressed.

Differential expression results were visualized using hierarchical clustering heatmaps generated with the ComplexHeatmap package. PCA and functional enrichment analyses (Gene Ontology and Kyoto Encyclopedia of Genes and Genomes pathways) were performed using ggplot2 and clusterProfiler packages in R. Venn diagrams were generated online using InteractiVenn (https://www.interactivenn.net/).

### Accession numbers

Sequence data from this article can be found in the NCBI database BioProject ID PRJNA1307209.

## Supplementary Material

kiaf463_Supplementary_Data

## Data Availability

All raw RNA-seq data generated in this study can be found at the NCBI Sequence Read Archive (BioProject ID PRJNA1307209), under the following accession numbers: SRR35172059, SRR35172060, SRR35172061, SRR35172062, and SRR35172063. The full RNA-seq workflow, including input files, differential expression code using edgeR, plots, and documentation, are available at https://github.com/Barbara574/RNAseq_Qlobata. Proteome sequencing data are available in the PRIDE database under project accession PXD066721.

## Supplementary data

The following materials are available in the online version of this article.

**Supplementary Figure S1.** PCA plots of the proteome and transcriptome datasets from the acorns produced in aCO_2_ and under eCO_2_.

**Supplementary Figure S2.** Bubble plots illustrating the GO term enrichment analysis of decreased differentially abundant transcripts and protein.

**Supplementary Table S1.** MS-based identification and quantification data for all proteins detected in acorns aCO_2_ and eCO_2_ conditions.

**Supplementary Table S2.** List of DEPs showing fold change (logFC) ratios (eCO2/aCO2) and *q*-value (Benjamini–Hochberg method) is included.

**Supplementary Table S3.** List of DEGs under eCO_2_ compared to aCO_2_.
