# Supplementary material for: Elevated atmospheric CO2-induced reprogramming leads to decreased seed protein and nutritional quality in forest trees
Source: Plant Physiol. 2025 Sep 30;199(3):kiaf463. doi: 10.1093/plphys/kiaf463 (PMC12596716; doi:10.1093/plphys/kiaf463)
Supplement: kiaf463_Supplementary_Data [file kiaf463_supplementary_data.zip › PLPHYS-2025-0909R1_Supplementary Figures.pdf]

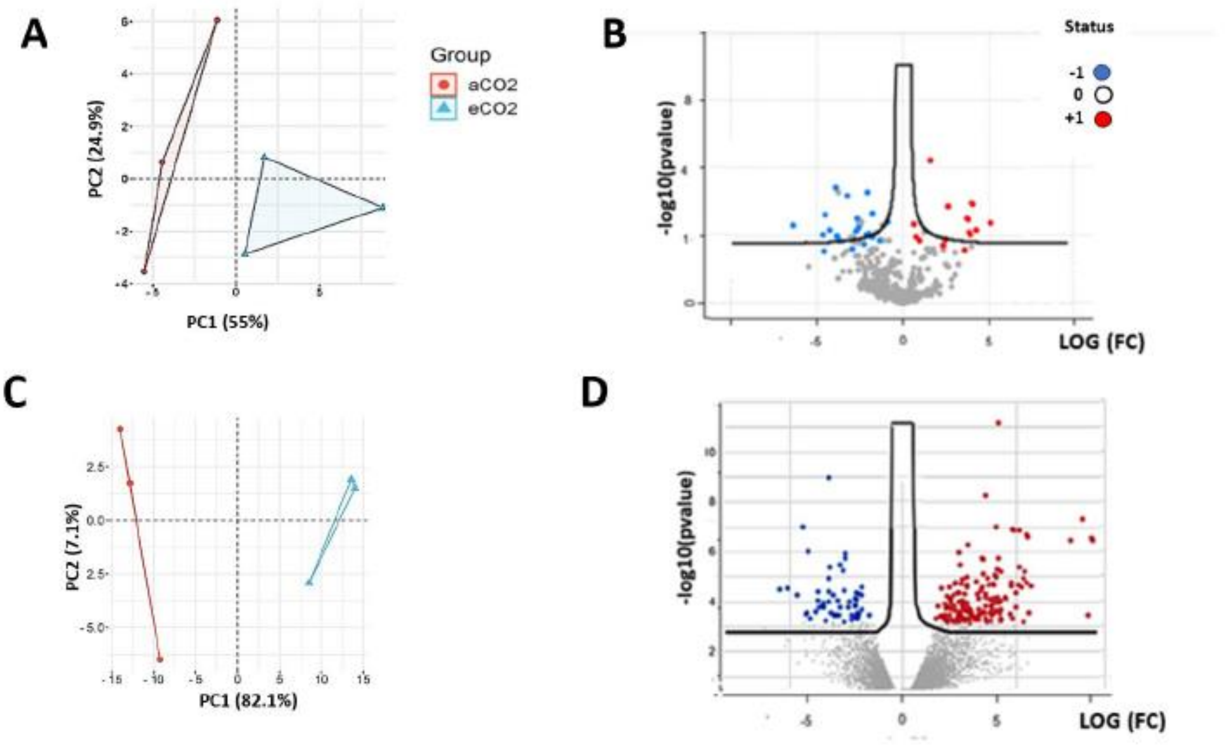

Supplementary Figure S1. Principal component analysis (PCA) plots of the proteome and transcriptome datasets from the acorns produced in air (aCO_2) and under elevated CO_2 (eCO_2). The plots show the similarities between the two groups of samples (aCO_2 and eCO_2) in the proteome A and transcriptome (C) data sets. The Volcano plots show the significance and log-fold-change for differentially expressed proteins (B) and transcripts (D) expressed as the ratio of values under eCO_2 compared to aCO_2.

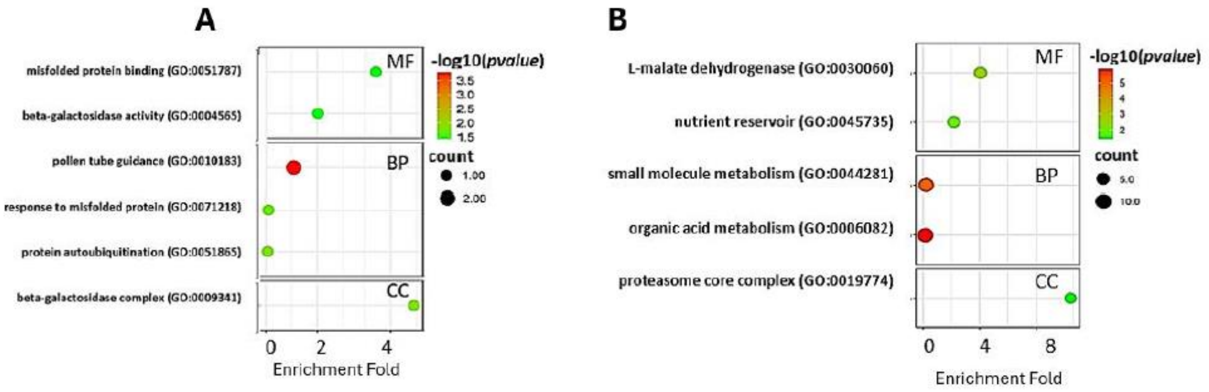

Supplementary Figure S2. Bubble plots illustrating GO term enrichment analysis of decreased differentially abundant transcripts and protein.

The "Count" represents the number of differentially expressed transcripts (DEGS; A) and proteins (DEPs; B) associated with each GO term. The Enrichment Fold indicates the ratio of enriched DEPs or DEGs to the total background genes/proteins. The p-value was corrected using the Benjamini-Hochberg (BH) method.

Examples of DEPs and DEGs for each category; **MF-molecular function** (DEPs) A0A7N2MBX6 A0A7N2RBQ4 A0A7N2LXI9 -malate dehydrogenases, (DEGs) LOC115952336, LOC115984392- beta galactosidases, **BP- biological processes** (DEPs)-A0A7N2KTK4, A0A7N2R0B3 -response to missfolded protein, (DEGs) LOC115982390, LOC115969897- organic acid metabolism, **CC-cellular component- (**DEPs) beta galactosidase complex, (DEGs) proteasome complex.
